# Employing cofacilitation to balance power and priorities during health service codesign

**DOI:** 10.1111/hex.13875

**Published:** 2023-09-19

**Authors:** Reema Harrison, Bronwyn Newman, Ashfaq Chauhan, Mashreka Sarwar

**Affiliations:** ^1^ Australian Institute of Health Innovation Macquarie University Sydney New South Wales Australia

## 1 INTRODUCTION

Applications of codesign and related participatory approaches endeavour to democratise the process and product of health service research and improvement efforts. As a group of methods that are ground in a user‐centric, participatory approach, codesign is valued for supporting people with lived experiences of a health condition or service to have their voices heard and acted upon via their contributions to codesign activities.^1^, ^2^ It is now well‐established that the practice of codesign is fraught with challenges in ensuring equity, access to and inclusion in its processes to realise its proposed gains.^1^, ^2^, ^3^, ^4^ Those who facilitate codesign activities, by planning, guiding and supporting the codesign process, are central actors who, through their practice, seek to achieve a democratic, effective and inclusive process.

Yet codesign facilitators are a heterogenous group of individuals who may occupy a variety of additional roles relevant to the codesign goals beyond that of facilitating the process. Facilitators' background, personal characteristics, motivations or other attributes may influence the group dynamic and ultimately outcome. In the context of health services research and improvement activities, codesign facilitators are commonly academic researchers, clinicians and health service staff who are invested in the outcome from the process.^5^ In this article, we consider the role of facilitators in the process and outcomes of codesign of health services research and improvements. Drawing upon our experiences in health services codesign with diverse communities, we explore the potential and pitfalls of cofacilitation with consumers as a strategy to address power differentials between, and the differing priorities of, facilitators and codesign members. Ultimately, we propose guiding principles for those who seek to engage in cofacilitation in their codesign practice. We reflect on our experiences from the CanEngage Project as a case example to illustrate how these principles of cofacilitation in codesign were applied in practice.

### 1.1 Effective facilitation is fundamental to achieving the proposed gains of codesign

In recent years, strategies to manage power imbalances and create democratic and inclusive processes that reflect members' priorities have been detailed in a multitude of articles.^2^, ^6^ The strategies described often hinge on the planning of codesign work to build rapport and ensure respectful interactions, but also make reference to facilitation practice during the codesign process.^6^, ^7^ Effective facilitation can promote partnership and provide avenues for reciprocity, active participation, power distribution in decision‐making and shared decision‐making; elements of codesign that form a foundation for inclusion.^8^, ^9^ Through facilitation, opportunities to enable and affirm the value of diverse contributions are created for codesign members. For example, using inclusive language, limiting jargon, inviting diverse opinions, and managing contributions to ensure equal opportunity to voice opinions.

Whilst engaging an impartial and experienced facilitator is often the preferred approach for effective codesign, applications of codesign in the public healthcare sector rarely afford this. Health service improvement activities are often driven by motivated individuals or rapidly advancing strategic changes; their practice is restricted by budget and timeline. As a result, professional experts (clinicians and health service researchers) are often assuming the role of facilitator. A degree of workflow knowledge in the facilitator is also often required to ensure that codesigned change aligns with and can be implemented in practice. Healthcare staff bring this professional expertise and often find themselves as codesign facilitators. Similarly, the desire for implementation and testing of interventions rather than design work in many research funding schemes means that health services researchers are often charged with the task of facilitating codesign of interventions or programs within the budgetary and time constraints of preliminary work. Health services researchers also apply their professional expertise to create alignment between proposed codesigned change and contemporary theory, models and existing interventions. Although commonplace, professional experts assuming facilitation role has implications for power imbalance that may inadvertently drive the codesign priorities and the process that ensues, with the potential to reduce the voice of consumer members.

### 1.2 Cofacilitation in codesign

One approach that has sought to redress these imbalances is the use of cofacilitation, in which two or more facilitators who represent the cohorts of codesign members present work together to manage the group process. Cofacilitation has been utilised for some time in it embodies participatory work across a range of health settings and may further be applied to redress power imbalances in the context of codesign.^10^ For example, in a health service improvement project in which both clinicians and consumers are contributing, the codesign may be cofacilitated by a clinician and a consumer facilitator working together. Such approaches seek to leverage the skills and perspectives of each cofacilitator to create a dynamic and balanced approach to facilitation. Central advantages that we have observed of this approach are (i) greater ability of two or more facilitators to observe and act upon shifts in the power dynamic, problems and recognise issues that require attention and (ii) creation of a more inclusive culture to encourage engagement by incorporating diverse perspectives in the facilitation team. Through cofacilitation, each facilitator may be supported to be accountable for their practices and approach and engage in reflexivity regarding their role and positionality in the codesign process. Reflective practice can be used to regulate their role and create distance between their professional expertise, personal experiences, priorities and the group process and outcomes.

**Figure 1:**
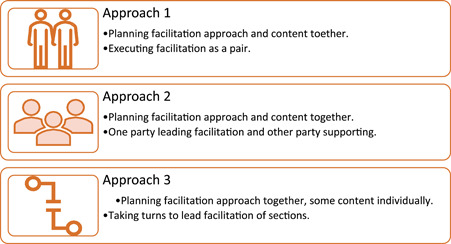
Approaches to cofacilitation.

Figure 1 describes three main approaches that we propose may be employed by cofacilitators to conduct codesign work. In Approach 1, cofacilitators work together to design, plan and execute the workshops in a dynamic and collaborative process. In Approach 2, one facilitator takes the lead role and the other/s a supportive role. The lead facilitator guides the process and is supported by the other by managing a large group, subsets of the group or specific activities. This approach is best suited for large group sessions, or those that involve multiple or complex activities. In Approach 3, facilitators take an alternating lead role; each may plan a section of the session and lead that element, supported by their cofacilitator if required. Rather than being mutually exclusive, cofacilitators may engage in these approaches on a continuum as we have found during our codesign practice.

Collectively as authors we have recently engaged in cofacilitation using the first of these approaches in the CanEngage Project.^11^ CanEngage is a 4‐year, multisite project, which has sought to increase healthcare safety outcomes for people from diverse cultural and linguistic backgrounds accessing cancer care. Codesign was central to the research, with several discrete codesigns being conducted to create change for improvement at hospitals in two states of Australia. In each codesign, we used cofacilitation between clinician–researchers and consumers (including the authors) from diverse cultural and linguistic backgrounds. Each codesign workshop series focussed on codesigning a strategy to increase the safety of cancer care for the community at a single service. A diverse group of cancer care service users were recruited and trained in cofacilitation at the outset of the project; the CanEngage Network.^12^ Members were informed about the project aims and a guide for cofacilitators was developed collaboratively.

For each codesign, a consumer cofacilitator worked with a clinician–researcher throughout the process of planning the workshop, creating appropriate content and cofacilitating the workshops. The cofacilitators met regularly before and between the workshops to reflect on the process and plan the next stage. Facilitation roles during the codesign workshops were negotiated when planning each workshop, with primacy on the consumer cofacilitators comfort in relation to their role/s. Facilitation roles included supporting interaction in break‐out groups, time keeping, coordinating group activities and facilitating contact with consumers informally to build rapport and confidence to interact in the workshop setting.

Through initial conversations about our approach to facilitation together, we endeavoured to adopt an approach that reflected Approach 1 at the outset of our process, yet we ultimately utilised an approach that was more comparable to Approach 3. Reflecting on the collective workshops undertaken, workshops were designed and planned together, but consumer facilitators generally expressed a preference to deliver planned facilitation activities for specific sections of the workshop. Consistently, consumer facilitators assumed a peer‐support role with consumers in and between codesign workshops that provided an opportunity to support diverse contribution. Applying Approach 3 also provided a stronger structure for the clinician–researcher cofacilitators because it provided clear boundaries for them in executing the workshop and managing their contribution to the facilitation. Having such boundaries meant that they could provide leadership and support at planned points in the process, but not assume the leadership role throughout.

### 1.3 Guiding principles for cofacilitation in codesign practice

Despite recognition of cofacilitation as a approach for guiding group processes, few have sought to describe the practicalities of this and specifically in the context of codesign.^1^, ^3^ Drawing upon our experiences in CanEngage, we propose the following 10 practical suggestions for navigating cofacilitation practice to balance power and priorities to support equitable contributions in codesign practice:

1. Relationship establishment—at the outset of the relationship, reflexivity and acknowledgement of the background, skills, personal circumstances and experiences of facilitator and how this may impact your work together is critical. For example, facilitators' past experiences of the topic but also of involvement in research and/or facilitation, personal circumstances and supports required to facilitate with confidence. Recognition for consumers, both financial and nonfinancial, should also be agreed.
2. Role definition and distinction—establish and mutually agree how the role of facilitator is defined and how this will look for each facilitator. Agree the extent of flexibility in role sharing, how this will be negotiated, and the approach should there not be consensus. Facilitators may develop a living document with this information to revisit throughout their relationship.
3. Setting expectations—discuss what each party expects from the cofacilitation and codesign process, how they expect to contribute, what preparation and training might be valuable, and how they expect others to contribute—seeking to align expectations where possible.
4. Openness and debriefing—establish mechanisms for open discussion and opportunities to regularly debrief informally and formally throughout the relationship and process of codesign, including in evaluating the cofacilitation process at its end.
5. Communication signals—identify how communication will occur between facilitators during codesign sessions for example to indicate the need to pause, regroup, change course or extend focus into particular areas. A brief post‐it note from one facilitator to another was often used during CanEngage sessions for this purpose.
6. Practice sessions—undertake several practice sessions that may unfold in different ways to enable facilitators to test out agreed approaches and identify opportunities to improve, add or rethink approaches for in‐session interaction. CanEngage practice workshops often led to changing the role or sections each facilitator would lead on.
7. Support and respect—before, during and after co‐facilitation practice maintain a supportive and respectful approach to collaborating by using the agreed mechanisms to identify areas of concern and adhere to the agreed approaches for cofacilitation practice throughout the relationship from the outset and as these approaches evolve.
8. Check in during sessions—establish an opportunity to check in during sessions as part of the session structure to provide an avenue to tackle emerging issues and your cofacilitation strategy. For example, during the CanEngage Project, facilitators would discuss the group dynamic briefly at the start of morning tea and whether any changes to our plan were needed.
9. De‐brief and feedback after sessions—formal opportunities for debriefing and providing reciprocal feedback on one another's practice built into each engagement provide an avenue for deliberate reflection, when possible, immediately and then some time after the session.
10. Sharing tasks between meetings—a practical expression of power flattening between cofacilitators is deliberate sharing of tasks to plan for the subsequent session between meetings.

## 2 CONCLUSION

Cofacilitation offers an opportunity to address professional and power imbalances during codesign in health care, which is critical in the context of clinicians and academics frequently assuming facilitation roles. Yet navigating cofacilitation is challenging in practice, with limited guidance available. Based on our experiences in utilising cofacilitation between consumers and clinician–researchers, we propose several practical suggestions for creating and sustaining and effective cofacilitation approach.

## AUTHOR CONTRIBUTIONS

All authors collaboratively conceived the manuscript concept and contributed ideas to the initial draft. Reema Harrison synthesised contributions into a first draft, with all authors contributing to the final draft.

## CONFLICT OF INTEREST STATEMENT

The authors declare no conflict of interest.

## PATIENT OR PUBLIC CONTRIBUTION

This piece of work is coauthored by a healthcare consumer from an ethnically diverse background who has lived experience of cancer that is a consumer co‐facilitator for codesign work in the CanEngage Project. The CanEngage Project described includes consumer investigators who conceptualised, designed and have been centrally involved in the execution of the project over 4 years, along with a cofacilitator network of nine culturally and linguistically diverse consumers who collaborate with clinicians and academics in conducting the research project.
